# Abdominal Massage for Adults With an Intellectual Disability and Constipation: A Feasibility Pilot Study

**DOI:** 10.1111/jar.70207

**Published:** 2026-03-11

**Authors:** Janet Finlayson, Kirsteen Goodman, Jennifer Crockett, Sudhakar Sharma, Josephine Kelly, Ceri Sellers, Suzanne Hagen

**Affiliations:** ^1^ Department of Nursing, Community and Public Health School of Health and Life Sciences, Glasgow Caledonian University Glasgow UK; ^2^ Community Learning Disability Physiotherapy Service NHS Greater Glasgow and Clyde Glasgow UK; ^3^ Community Learning Disability Physiotherapy Service NHS Lanarkshire Bothwell UK

**Keywords:** abdominal massage, bowel management, constipation, intellectual disability, non‐pharmalogical intervention

## Abstract

**Background:**

Between 33% and 50% of adults with an intellectual disability experience constipation. The predominant treatment of laxatives is poorly managed and lacks efficacy over time.

**Aim:**

The aim was to determine the feasibility of, and pilot, abdominal massage for adults with an intellectual disability and constipation, administered by their supporter at home, to alleviate their symptoms.

**Method:**

Adults with an intellectual disability and constipation were randomised, using a 3:1 ratio, into intervention and control groups. Supporters of adults in the intervention group received training and completed abdominal massage sessions with the person over a 6‐week period.

**Results:**

Thirty‐one adults and their supporters participated: 24 intervention and 7 control groups. Abdominal massage was found to be a feasible non‐pharmacological intervention to use in this population.

**Conclusion:**

A large trial is required to determine the effectiveness of this intervention. Co‐production is also needed to develop tailored constipation symptoms assessment for this population.

## Lay Summary

- Adults with an intellectual disability experience high rates of constipation, but laxatives prescribed to alleviate constipation symptoms are poorly managed and lack efficacy over time.
- Home‐based abdominal massage is a feasible non‐pharmacological intervention for adults with intellectual disability and their supporters.
- There is an urgent need to co‐produce a constipation assessment tool with adults with an intellectual disability, their supporters and clinicians.

## 1 Introduction

Adults with an intellectual disability experience higher rates of physical and mental health conditions compared to adults without an intellectual disability in the general population (Liao et al. 2021). Multiple co‐morbidity and polypharmacy (McMahon et al. 2021; Liao et al. 2021) are problematic for adults with an intellectual disability, as co‐occurring health issues and medication interactions as well as side effects can further impact on the person's health and wellbeing.

Adults with an intellectual disability have a 50% increased risk of experiencing constipation, compared to the general population, mainly because of poor diet (lacking fibre), limited mobility (because low physical activity weakens bowel muscles leading to constipation) and medication side effects (Horan et al. 2024; Robertson et al. 2018). Constipation can be defined as a decreased frequency of bowel movements characterised by passing hardened stools, associated with straining, pain and possible episodes of overflow faecal incontinence, for more than 8 weeks (Drossman 2016). Constipation is categorised as being primary (with no underlying medical cause but associated with lifestyle), secondary (caused by health conditions, such as cerebral palsy or diabetes) or iatrogenic (caused by medication side effects) (Public Health England 2016). Constipation is a leading co‐occurring morbidity associated with epilepsy, and the side effects of anticonvulsant medication add complexity to these co‐occurring issues (Laugharne, Wilcock, et al. 2024; Gabrielsson et al. 2023). The prevalence of epilepsy amongst adults with an intellectual disability is 25%, and higher for adults with a profound intellectual disability (Snoeijen‐Schouwenaars et al. 2021).

Constipation is a serious concern for adults with an intellectual disability. It is estimated that between 33% and 50% of adults with an intellectual disability experience constipation, with higher rates reported for adults with an intellectual disability with non‐ambulatory cerebral palsy and adults with a profound intellectual disability (Horan et al. 2024; Laugharne, Sawhney, et al. 2024; Robertson et al. 2018; Public Health England 2016). Adults with an intellectual disability and constipation are more likely to experience emergency hospital admissions, and in extreme cases individuals with an intellectual disability can die as a result of faecal impaction (Roberts et al. 2023; Public Health England 2016).

Being prescribed laxatives is the predominant management response to constipation for people with an intellectual disability—and the general population—yet the reliance on laxatives is often poorly managed and ineffective over time (Bishop et al. 2024). Within health and social care services for adults with an intellectual disability in the United Kingdom and Ireland, as well as laxative use, constipation management and treatment can include promoting an adequate diet (fibre) and fluid intake, mobility, exercise, a toileting plan, optimal positioning when sitting on a toilet, education for adults with an intellectual disability and their supporters (relatives or support workers), health and medication review, and non‐pharmacological interventions, such as biofeedback or abdominal massage (Horan et al. 2024; Robertson et al. 2018; Public Health England 2016). Abdominal massage is a non‐invasive technique which involves massaging a person's stomach area (manually or using a device) in a clockwise fashion for 10–20 min to stimulate peristalsis, thus reducing colonic transit time and increasing the frequency and ease of bowel movements (McClurg et al. 2020).

Previous research on abdominal massage for people with an intellectual disability is limited but encouraging. Emly et al. (1998) investigated abdominal massage over 7 weeks with 32 adults with a profound intellectual disability who lived in an institution, compared to a laxative regime for 7 weeks. They did not find any measurable difference in outcomes between the two interventions over 7 weeks assessment periods, but they did find that abdominal massage produced positive outcomes in the adults being more tolerant of touch, and improvements in their behaviour and communication. A study conducted with parents of 25 children with an intellectual disability, reported a number of positive quality of life outcomes for their children following abdominal massage; mainly relief from constipation symptoms (reported 88% of the children), less laxative use (58%) and improvements in the child's dietary intake (41%) (Bromley 2014). Connor et al. (2014) also found, from their survey of abdominal massage being implemented for adults with an intellectual disability by a community‐based healthcare team in England, United Kingdom, that family carers and supporters found abdominal massage easy to learn and implement. They also found that abdominal massage outcomes being reported were mainly improved changes in bowel movements (e.g., texture and frequency), person experiencing less pain, less low mood, fewer behaviour/s that challenge and a reduction in laxative use. The aim of this study therefore, was to pilot and determine the feasibility of evaluating abdominal massage as an intervention for adults with an intellectual disability and constipation, administered by their supporter at home. A feasibility pilot study is important for establishing whether the intervention is acceptable (e.g., sample recruitment and retention), and that the methods are appropriate, before proceeding to a larger clinical trial.

## 2 Method

### 2.1 Participant Recruitment and Sample

Community Intellectual Disability Team (CLDT) health professionals across (National Health Service Greater Glasgow and Clyde and NHS Lanarkshire), were asked to provide project information to adults with an intellectual disability on their caseloads who met the inclusion criteria. The inclusion criteria was: adults with an intellectual disability known by their health care team to experience constipation, including those who are prescribed laxatives but still prone to constipation, those who use enemas but still have problems emptying their bowels, and those who have been previously been admitted to hospital due to impaction. Adults with an intellectual disability with the following issues were excluded: history of a malignant bowel obstruction or abdominal growth; history of inflammatory disease of the intestine (e.g., Crohn's disease); recent abdominal surgery or scarring or skin lesions; abdominal hernia; pregnancy; indwelling catheter; and has had abdominal massage within the last 3 months. Adults with an intellectual disability and their supporters who were interested in taking part, after reviewing the information about the project they received, were asked to contact the research team directly to take part.

Forty‐three adults with an intellectual disability and constipation were eligible to take part in this study, but seven were excluded because of nearest relative or welfare guardian consenting issues (e.g., no nearest relative or welfare guardian identified for the person) (Scottish Government 2000). A further five adults and their supporters expressed interest in taking part but could not according to the study's exclusion criteria; two adults for medical reasons; and three adults because they had abdominal massage within the previous 3 months. Thirty‐one adults with an intellectual disability did take part in this study, with support from their supporter (e.g., relative or support worker), which was 78% of our target sample size of 40 adults. These participants were randomised into either an intervention or control group using a 3:1 ratio, respectively. The target number of 10 controls was cautious due to adults with an intellectual disability and their supporters being potentially hard to recruit, and sensitivity to participant burden (10 was sufficient to determine whether participants could be recruited as controls) (Nay et al. 2024). Randomisation was carried out by an independent statistician, employing block stratification, to balance the numbers in each group based on level of intellectual disability (mild, moderate, severe or profound). The sample characteristics are presented in Table 1.

**TABLE 1:** Sample characteristics.

	Whole sample (*N* = 31)	Intervention group (*N* = 24)	Control group (*N* = 7)
Age (years)	Median 32	Median 33	Median 26
	Range 19–63	Range 20–63	Range 19–38
Sex			
Male	19	15	4
Female	12	9	3
Ethnicity			
White British	29	22	7
Asian/Asian British	1	1	0
Other white background	1	1	0
Intellectual disability level			
Mild	2	2	0
Moderate	6	5	1
Severe	10	6	4
Profound	13	11	2
Cause of intellectual disability			
Unknown	17	15	2
Down syndrome	4	1	3
Brain damage (e.g., bleed)	3	2	1
Birth injury	1	1	0
Premature birth	1	1	0
Alexander disease	1	1	0
SCN8	1	1	0
West syndrome	1	1	0
Rhetts syndrome	1	1	0
Lennoxgastut syndrome	1	0	1
Accommodation type			
Lives family	18	14	4
Supported living	13	10	3
Mobility level			
Independent	8	6	2
Requires support	1	1	0
Wheelchair dependent	22	17	5
Toileting			
Independent toileting	3	2	1
Needs toileting support	28	22	6
Health conditions			
Epilepsy	23	20	3
Neuromotor issues	22	17	4
Gastrointestinal issues	20	15	5
Musculoskeletal issues	18	14	4
Visual impairment	18	12	6
Behaviours that challenge	18	13	5
Respiratory issues	15	13	2
Sensory processing issues	15	13	2
Cerebral palsy	13	10	3
Skin condition	11	10	1
Mental health issues	10	8	2
Autism	7	6	1
Neurological (e.g., stroke)	4	3	1
Kidney disease	3	2	1
Hearing impairment	3	2	1
Diabetes	2	2	0
Heart disease	2	1	1
Dementia	1	1	0

Number of prescribed drugs	Median 10	Median 11	Median 8
	Range 2–20	Range 3–18	Range 2–20
Prescribed laxatives?			
Yes	24	18	6
No	7	6	1
Moderate exercise per week			
< 30 min	28	23	5
30–90 min	2	0	2
90–150 min	1	1	0
> 150 min	0	0	0

The target sample size of 40 participants with an intellectual disability, based on a 3:1 randomisation, was in part influenced by time and budget restraints. However, in this study, the target sample size and unequal randomisation was acceptable to maximise information on implementation and accessibility of the intervention for adults with and intellectual disability and their supporters, minimising participant burden, recruitment and retention and patient safety through detection of adverse events (Hopewell et al. 2025; Kunselman 2024; Nay et al. 2024).

### 2.2 Process and Materials

Project information, consent forms and interview questionnaires were developed by the research team for the purpose of this study, with input from the project's advisory team. Advisory team members were recruited via the research teams own networks. Parents of people with an intellectual disability were members of the advisory team, and their relatives with an intellectual disability contributed indirectly via their parents. These project materials were accessible, in that they used pictures, symbols and easy language for use with adults with an intellectual disability.

All participants were visited at home by a research physiotherapist (National Health Service Greater Glasgow and Clyde and NHS Lanarkshire), with support from their supporter, to complete the consent process and answer questions to collect demographic and health information.

All participants, with support from their supporters, were asked to complete a 7‐day bowel diary on two separate occasions: prior to commencing the 6‐week assessment period, and at the end of the 6‐week period. The bowel diary recorded the following information: frequency of defecation; stool type using the Bristol Form Stool Scale (BFSS) (Probert et al. 1993); laxative use; and any instances of faecal smearing/incontinence.

All participants, with support from their supporter, were also asked to complete the Knowles‐Eccersley Symptom Score (KESS) (Knowles et al. 2000) at both points in time, which is a validated questionnaire used in clinical settings to assess constipation. KESS produces a score rating of either not being constipated (score between 0 and 10) or being constipated (score between 11 and 38).

Participants with an intellectual disability, and their supporters, were who were randomly assigned to the intervention group, were given a choice of completing manual abdominal massage or abdominal massage using a device over a 6‐week assessment period, based on their preference and lifestyle. The abdominal massage device used in this study, was MoWOOT (https://www.mowoot.com/en/). This device was considered safe to use and effective for the treatment of constipation (McClurg et al. 2020). The intervention group's support teams were then given training on either manual or device abdominal massage, based on their choice, over one session by the research physiotherapist. The abdominal massage training comprised one practical session, video and manual. The manual abdominal massage video and manual were developed by National Health Service Greater Glasgow and Clyde and NHS Lanarkshire Community Intellectual Disability Physiotherapy Service, and adapted for this study. The abdominal massage using a device training video and manual was adapted from resources available on the MoWOOT website (https://www.mowoot.com/en/). Participants and their supporters were advised that the recommended schedule would be 15 min per day for manual abdominal massage, or 20 min per day for abdominal massage using a device, at least 5 days per week over the 6‐week assessment period.

Both intervention and control groups' participants received telephone support from the research physiotherapist on a weekly basis during the 6‐week assessment period.

Participants with an intellectual disability, who were randomly assigned to the control group, continued with routine care only during the 6‐week assessment period.

At the end of the 6‐week period, all participants completed a follow up questionnaire, and a sub‐sample of supporters of adults with an intellectual disability in the intervention group was asked to evaluate their abdominal massage training.

### 2.3 Analysis

IBM SPSS version 24 was used to generate frequency and descriptive results. Statistical analysis was not conducted due to the small sample size. Qualitative data collected via open‐ended questions in the questionnaires was subject to thematic and content analysis (Braun and Clarke 2021).

### 2.4 Ethics Statement

This study was conducted with adults across all levels of intellectual disability (mild to profound), and their supporters. Ethics approval was granted by the national Scotland A Research Ethics Committee. In keeping with the Adults with Incapacity (Scotland) Act 2000 (Scottish Government 2000), all adults with an intellectual disability participated as much as they were able to, and either gave their own consent to take part, or their nearest relative or welfare guardian/attorney consented with them on their behalf.

## 3 Results

### 3.1 Sample

The majority of adults in this sample had a severe or profound intellectual disability (23 out of 31 participants), with a high level of support needs. For example, only eight adults had no mobility issues, and only three adults did not require support for toileting. The most commonly reported health condition, following constipation (31 adults), was epilepsy (28 adults). Polypharmacy was common (median number of prescribed drugs was 10 drugs), and 24 (77%) adults were being prescribed laxatives for their constipation.

Twenty‐four adults with an intellectual disability were randomly assigned to the intervention group, and seven adults with an intellectual disability were randomly assigned to the control group.

### 3.2 Compliance and Usability

Nineteen adults assigned to the intervention group and their supporters chose abdominal massage using a device, and five adults and their supporters chose manual abdominal massage. Out of these 24 participants, two participants withdrew after randomisation but pre‐intervention. A further two of these participants withdrew during the intervention period: one person because they could not accept the MoWOOT device being used with them, and one person for a physical reason not related to abdominal massage (see next section on ‘Adverse Events’).

Overall, compliance with abdominal massage was high for 14 out of the remaining 20 participants in the intervention group (reported via weekly telephone calls), but only six out of these participants complied with the whole recommendation of 15/20 min of abdominal massage at least 5 days per week over the 6‐week assessment period. The reasons given for non‐compliance with their abdominal massage regime were as follows: illness (five adults); refusal (four adults); not available (e.g., visiting parents or a respite centre) (four adults); behaviour that challenges (one adult); and epileptic seizure (one adult). More than one reason was given for one of these adults.

At the end of the study period, 15 participants and their supporters intended to continue their abdominal massage sessions. Three discontinued because they did not like/tolerate abdominal massage, and two did not because they had not found abdominal massage to be beneficial. All participants who had been using a MoWOOT device during the study period were able to keep their device at the end of the study.

### 3.3 Adverse Events

Six adults with an intellectual disability in the intervention group experienced adverse events during the study period. Four adults experienced a respiratory infection, which interrupted their abdominal massage schedule for 1 week. One adult was hospitalised for a gastric feeding issue, which interrupted their abdominal massage schedule for 3 weeks, and one adult experienced a physical issue (unsteadiness), which led to their withdrawal from the intervention after 3 weeks.

### 3.4 Abdominal Massage Training

Seventy support team members of 24 adults with an intellectual disability in the intervention group (between one and eight supporters per participant) received abdominal massage training. All 22 supporters from a sub‐sample who were asked to evaluate training on abdominal massage reported that one practical demonstration was enough, and that the training provided sufficient information on abdominal massage for them to feel confident in applying it.

### 3.5 KESS

Participant responses for each item on the KESS at baseline (pre‐intervention) are presented in Table 2 for the whole sample of 31 adults with an intellectual disability, as well as baseline and 6‐week responses for the intervention and control groups. Data is missing for 6 out of 11 items in the questionnaire because participants and their supporters either found the questions too difficult to understand (wording) or too subjective; in that the adult with an intellectual disability could not answer the question, and their supporter could not answer for them on their behalf. In addition, question 10 was not relevant to those in the sample who did not defecate in a toilet and relied on pads for continence management. This meant that KESS scores, which rely on complete data, could not be calculated for the majority of the sample.

**TABLE 2:** KESS results and scores.

Question (score rating)		Whole sample	Intervention group		Control group	
		Baseline *N* = 31	Baseline *N* = 24	6‐week follow up *N* = 20	Baseline *N* = 7	6‐week follow up *N* = 7
1	Duration of constipation					
	0–18 months (0)	4	2	1	2	1
	18 months to 5 years (1)	5	5	6	0	0
	5–10 years (2)	6	5	2	1	1
	10–20 years (3)	2	1	0	1	1
	> 20 years (4)	14	11	11	3	4
	Missing	0	0	0	0	0
2	Laxative use					
	None (0)	2	2	5	0	1
	Occasionally, short duration (1)	6	5	0	1	1
	Regular, long duration (2)	22	17	13	5	5
	Long duration, ineffective (3)	1	0	2	1	0
	Missing	0	0	0	0	0
3	Frequency of bowel movement					
	1–2 times/1–2 days (0)	22	15	20	7	7
	2 or less times/week (1)	7	7	0	0	0
	Less than once per week (2)	2	2	0	0	0
	Less than once per 2 weeks (3)	0	0	0	0	0
	Missing	0	0	0	0	0
4	Unsuccessful evacuation attempts					
	Never/rarely (0)	7	6	14	1	4
	Occasionally (1)	11	8	2	3	1
	Usually (2)	8	6	0	2	0
	Always: manual evacuation (3)	0	0	0	0	0
	Missing	5	4	4	1	2
5	Feeling incomplete evacuation					
	Never (0)	1	1	1	0	1
	Rarely (1)	4	2	4	2	1
	Occasionally (2)	7	4	2	3	0
	Usually (3)	4	4	0	0	0
	Always (4)	0	0	0	0	0
	Missing	15	13	13	2	5
6	Abdominal pain					
	Never (0)	3	3	10	0	0
	Rarely (1)	9	6	1	3	2
	Occasionally (2)	10	9	4	1	1
	Usually (3)	2	1	0	1	1
	Always (4)	2	2	0	0	1
	Missing	5	3	5	2	2
7	Bloating					
	Never (0)	6	6	6	0	0
	Perceived by patient only (1)	1	0	1	1	0
	Visible to others (2)	19	14	13	5	6
	Severe: satiety or nausea (3)	3	3	0	0	1
	Severe with vomiting (4)	2	1	0	1	0
	Missing	0	0	0	0	0
8	Enemas/digitation					
	Never (0)	15	11	14	4	5
	Enemas/suppositories occasionally (1)	11	9	5	2	1
	Enemas/suppositories regularly (2)	5	4	1	1	1
	Manual evacuation occasionally (3)	0	0	0	0	0
	Manual evacuation always (4)	0	0	0	0	0
	Missing	0	0	0	0	0
9	Time taken (minutes in lavatory/attempt)					
	< 5 min (0)	6	4	5	2	1
	5–10 min (1)	6	4	4	2	0
	10–30 min (2)	5	4	2	1	4
	> 30 min (3)	8	6	0	2	0
	Missing	6	6	9	0	2
10	Difficulty evacuation (causing painful evacuation effort)					
	Never (0)	8	6	15	2	3
	Rarely (1)	7	4	0	3	1
	Occasionally (2)	4	4	2	0	1
	Usually (3)	4	3	0	1	0
	Always (4)	2	2	0	0	0
	Missing	6	5	3	1	2
11	Stool consistency (without laxatives)					
	Soft/loose/normal (0)	3	3	4	0	1
	Occasionally hard (1)	9	6	0	3	1
	Always hard (2)	6	4	4	2	0
	Always hard, usually pellet‐like (3)	2	1	2	1	1
	Missing	11	10	10	1	4
Total score						
Not constipated (score 0–10)		1	0	1	1	0
Constipated (11–39)		10	6	2	4	1
Missing		20	18	17	2	6

Complete data at baseline and 6‐week follow up was only available for one participant in the intervention group (scoring 14 constipated and 4 not constipated, respectively), and one participant in the control group (scoring 15 constipated and 16 constipated, respectively).

### 3.6 Bowel Charts

All participants in the intervention and control groups were able, with support, to complete a bowel diary for at least 5 days at baseline and post‐intervention. These results are presented in Table 3.

**TABLE 3:** Bowel chart data.

	Baseline		Post‐intervention	
	Intervention group (*N* = 20)	Control group (*N* = 7)	Intervention group (*N* = 20)	Control group (*N* = 7)
Person passed a stool at least once per day	3	2	5	5
Number of days no stools passed	3 (IQR* 3)	1.5 (IQR 2)	1.5 (1QR 3.5)	0 (IQR 1)
Stool type (ranging from 1 = separate hard lumps to 7 = extremely liquid)	5 (IQR 2)	6 (IQR 3.5)	5 (IQR 2)	4 (IQR 3)

* Interquartile range.

### 3.7 Laxative Use and Going for a Poo

Results for laxative use at baseline and post‐intervention period are presented in Table 4. This table also includes Likert scale results for the subjective question, ‘How do you feel about going for a poo?’

**TABLE 4:** Laxative use and going for a poo.

	Baseline		Post‐intervention	
	Intervention group (*N* = 20)	Control group (*N* = 7)	Intervention group (*N* = 20)	Control group (*N* = 7)
Average number of days prescribed laxatives in a week	5 (median 5)	4 (median 6)	6 (median 4.5)	7 (median 7)
Person being administered less laxatives than usual	1 (median 0)	1 (median 0)	0 (median 0)	0 (median 0)
Person being administered more laxatives	0 (median 1)	1 (median 1)	0 (median 0)	1 (median 1)
How the person feels about going for a poo (between 1 = very unhappy to 7 = very happy)	Median 4 (IQR 2)	Median 2 (IQR 3)	Median 5 (IQR 2)	Median 3 (IQR 3)

### 3.8 Self/Proxy Reported Outcomes

The 20 adults with an intellectual disability and their supporters provided their own comments about abdominal massage outcomes. These outcomes are listed in Table 5. The numbers do not add up to 20 because some participants reported more than one outcome.

**TABLE 5:** Self/proxy‐reported abdominal massage outcomes for intervention group.

Outcome	*N* = 20
Night time and sleep	
Person is sleeping better at night	2
Person is waking less during the night	2
Person is experiencing less flatulence at night	1
Daytime	
Person is more alert during the day	1
Bowel habits and constipation symptoms	
Person is passing stools more regularly	1
Person's stomach is less solid (but this makes it less obvious when they are needing to use the toilet)	1
Bowel movements are more regular now	1
Less straining/pushing to poo	1
Behaviour/s and distress	
Person has less behaviour that challenges	1
Person is less distressed	1
Wellbeing and comfort	
Person is happier/more content now	3
Person is more comfortable/experiencing less discomfort	2
Person is more relaxed	2
Improved wellbeing	1
Person is able to spend more time upright in their chair	1
Overall the person's life is better than it was before	1
Person looks forward to their abdominal massage sessions and enjoys them	1
Abdominal massage sessions have improved the person's trust in their support staff	1
Laxative use	
Less need for suppositories or enemas	3
Person is able to get out of the house more, instead of being stuck at home waiting for their suppositories to take effect	1
Health	
Does not have the same stomach problems as before	1
Fewer epileptic seizures	1
No change or difference	
No change	2

## 4 Discussion

The aim of this study was to pilot and determine whether home‐based abdominal massage administered by the person's supporter is a feasible intervention for adults across all levels of intellectual disability. Abdominal massage was found to be feasible, with the majority of participants and their supporters (19 out of 24 participants) choosing abdominal massage using a device to administer abdominal massage manually. Both options should be offered whenever possible, so the individual and their supporter/s can make the most appropriate choice for them. Six (32%) out of the 19 adults assigned to the intervention group experienced adverse events during the intervention period. These events were not related to abdominal massage but do demonstrate the complex health needs of adults with an intellectual disability. In line with previous research (Connor et al. 2014), the supporters of adults with an intellectual disability in this study found their abdominal massage training easy to learn over one session, and in addition, it led to them feeling confident in applying it.

In terms of outcome measures to evaluate the potential effectiveness of abdominal massage, we found bowel charts, laxative use records and self/reported outcomes to be useful to include in future studies. With regards to the former for example, all participants and their supporters in the intervention and control groups were able to complete bowel diaries for at least 5 days per week, at both baseline and post‐intervention. Previous studies on abdominal massage for people with an intellectual disability have utilised bowel charts (Bromley 2014; Emly et al. 1998), laxative use records (Bromley 2014; Emly et al. 1998) and self/proxy reported outcomes (Connor et al. 2014; Bromley 2014; Emly et al. 1998).

KESS, which is a valid and reliable measure of constipation symptoms across other patient groups, was not found to be suitable for use with adults across all levels of intellectual disability and their supporters in its current form; due to some questions being too difficult or too subjective to answer. In addition, inconsistent reporting was detected. One participant in the control group was reported to have been experiencing constipation for between 0 and 18 months at baseline, but for more than 20 years at 6‐week follow up (Table 2). Stool consistency could not be reported for 10 intervention group and between one and four control group participants, using KESS, despite daily bowel charts being completed with them throughout the assessment period. There is an urgent need to co‐produce and tailor a constipation symptom assessment tool with people with an intellectual disability, their supporters and clinicians.

The participants with an intellectual disability and their supporters self/proxy reported a total number of 22 positive outcomes following abdominal massage (Table 5), ranging from improvements in their sleep to a reduction in epileptic seizures and behaviour/s that challenge. This builds on previous research, which has also found abdominal massage for people with an intellectual disability to result in improvements in: sleep; touch tolerance; behaviour/s that challenge; communication; diet; as well as a reduction in laxative use and constipation symptoms; and less pain (Connor et al. 2014; Emly et al. 1998). Future research may consider transforming these into measurable outcomes by, for example, utilising sleep monitors, seizure charts and behavioural measures/assessments which have been developed for use with adults with an intellectual disability.

All 31 adults in the intervention and control groups were receiving routine NHS health care during the 6‐week assessment period, but we did not investigate this enough to establish what routine care entailed for these individuals in terms of constipation management and treatment. Whilst we did collect data about each person's laxative use, known health conditions, prescribed drugs and physical activity, we did not collect data about any other advice or treatment they may have been receiving, such as recommended changes to their diet and/or fluid intake or following an individualised toileting plan. It will be important to have a comprehensive understanding of this during future trials to be able to determine the potential effectiveness of abdominal massage in the context of overall constipation management and treatment (Horan et al. 2024).

### 4.1 Strengths and Limitations

The main strength of this study is that it evaluated the feasibility of abdominal massage training for supporters of adults with an intellectual disability, as well as abdominal massage as an intervention. The training materials used in this study, which were initially developed by NHS colleagues, are available directly from the authors.

Forty‐three adults with an intellectual disability and their supporters expressed interest in taking part in this study, but only 31 adults took part, which was 78% of our target sample size of 40 adults. Seven adults could not take part because of consent issues. This study highlights the issue of the lack of availability of a person's welfare guardian to consent on their behalf (when the person has no nearest relative) as a barrier to some adults with an intellectual disability with capacity to consent issues participating in research which has the potential to improve their health and wellbeing. Participant recruitment for this study was steady but slow over the participant recruitment period across two NHS sites. Future trials should consider longer periods of participant recruitment across multiple sites to ensure targets are met.

Seven adults with an intellectual disability and their supporters formed a control group, based on a 3:1 randomisation ratio. We included this in the study design to determine whether it was feasible to recruit control participants and their supporters, who were agreeable to receiving routine care only and not the abdominal massage intervention. Whilst we did find this to be feasible, a larger trial, adequately powered statistically, would need to include a control group based on a 1:1 randomisation ratio. In addition, the control group participants in this study were incentivised to remain in the study, as they were promised abdominal massage training at the end of the study period. All controls and their supporters took up the offer of training.

In this study, the advisory group was comprised of parents of people with an intellectual disability. Future studies should directly include adults with an intellectual disability as advisors.

## 5 Conclu‑sion

Home‐based abdominal massage, using either a device or administered manually, is a suitable non‐pharmaceutical intervention for adults with an intellectual disability and their supporters. A future trial is required to determine the potential effectiveness of this intervention in the context of overall constipation management and treatment. Co‐production of a constipation symptoms assessment tool is urgently required, to be able to understand, across clinical and home settings, the different types and symptoms of constipation adults with an intellectual disability experience, and also to develop reliable and valid measurable outcomes for future research.

## Author Contributions

J.F., K.G., J.C. and S.H. designed this research project and were responsible for day‐to‐day project management. S.S. and J.K. were expert advisors for this project. J.C. was responsible for data collection. C.S. was responsible for data entry. J.F., C.S. and K.G. conducted the data analysis. J.F. drafted the manuscript and all authors contributed towards the final version of the manuscript.

## Funding

This work was supported by the Baily Thomas Charitable Fund.

## Conflicts of Interest

The authors declare no conflicts of interest.

## Data Availability

The data that support the findings of this study are available on request from the corresponding author. The data are not publicly available due to privacy or ethical restrictions.
